# Editorial: Fetal/Embryonic Hematopoietic Progenitors and Their Impact on Adult Diseases

**DOI:** 10.3389/fcell.2021.732649

**Published:** 2021-08-18

**Authors:** Emanuele Azzoni, Charlotta Böiers, Silvia Brunelli, Antonella Ellena Ronchi

**Affiliations:** ^1^School of Medicine and Surgery, University of Milano-Bicocca, Monza, Italy; ^2^Division of Molecular Hematology, Lund Stem Cell Center, Lund University, Lund, Sweden; ^3^Department of Biotechnology and Biosciences, University of Milano-Bicocca, Milan, Italy

**Keywords:** hematopoieisis, macrophages, developmental biology, erythropoiesis, leukemia, EMP, DOHaD (development origins of health and disease)

Despite enormous progress boosted by significant technological advances of the last decade, a complete understanding of hematopoietic development has not yet been achieved. “Primitive” and “definitive” terms have been used for many years to describe the different waves of transient and persistent blood cells generated during ontogeny. However, it is now clear that this terminology is not adequate to address the surprising complexity of the developing hematopoietic system. Gaining additional insight into embryonic and fetal hematopoiesis will not only be relevant for textbook biology, but will carry important implications for the understanding of blood diseases, including genetic conditions and pediatric leukemia. This Research Topic comprises 14 articles including reviews and primary research articles focusing on different aspects on the biology of fetal blood cells, ranging from basic hematopoiesis to technological advances and translational studies.

## New Advances in Developmental Hematopoiesis: Increasing Layers of Complexity

An elegant review by Elsaid et al. delivers a comprehensive evolutionary perspective on hematopoietic development and shows that the concept of “layered” hematopoiesis is a successful conserved design introduced very early in evolution. Authors focus in particular on lymphoid cell development, which until few years ago was thought to exclusively derive from HSC activity. This view had implications in the readouts of *in vitro* or *ex vivo* experiments aimed at assessing the presence of HSCs; however, this concept has been recently challenged by the discovery of HSC-independent lymphoid progenitors within the embryo. Indeed, the fetal lymphoid compartment develops in sub-waves, which may, intriguingly, perform specific functions, similar to macrophages (see below).

A review from Neo et al. primarily focuses on mouse models and provides a useful and detailed overview of knockout and inducible fate mapping systems used to study hematopoietic development, which not only showcases its intricacies, but serves as a reminder of the importance of taking into account different and complementary experimental models when interpreting these data. The authors also review the specific functions of HSC-independent hematopoietic cells. Tissue resident macrophages (TRM) are derived from yolk sac erythro-myeloid progenitors (EMP), and exert a multitude of functions during development, including support for organogenesis, for vascular network formation and direct support for HSC development. Importantly, HSC-independent waves persist in the adult and perform unique functions, not limited to macrophages but also including mast- and lymphoid cells.

One of the reasons why developmental hematopoiesis is less studied than its adult counterpart is that cells of interest are not easily available. Indeed, research on human hematopoietic development has historically been hampered by the scarce availability of source material. For this reason, human pluripotent stem cells (hPCS) represent an important option, discussed by Gutierrez-Aguera et al. in a Perspective article contained in this Topic. These Authors provide an important comparison on the two most used hPSC hematopoietic differentiation protocols, which will be undoubtedly useful for many researchers in the field. For the same availability issue, single cell technologies were initially applied to the study of adult blood cells, but developmental hematopoiesis was soon to follow. Karlsson et al. here provide a timely review on how the advent of single cell technologies, which began with qPCR studies and followed up with transcriptomics, impacted and revolutionized developmental hematopoiesis, allowing to resolve cell heterogeneity and differentiation trajectories with unprecedented clarity.

## Macrophages: Key Players of Tissue Remodeling From the Embryo to the Adult

In their review Wu and Hirschi discuss the developmental origins of tissue-resident macrophages, describing how macrophages emerge along the three main waves of hematopoiesis, during primitive hematopoiesis, erythro-myeloid progenitor (EMP) generation, and definitive hematopoietic stem cell (HSC)-mediated hematopoiesis and how these stages were characterized by using different mouse models. Furthermore, they briefly outline macrophages molecular regulation in specific tissues and their impact on embryonic development and postnatal homeostasis, focussing in particular on angiogenesis, erythropoiesis, neurogenesis and osteogenesis.

On this last particular topic Yahara et al. review the heterogeneity and role of macrophages and osteoclasts during bone homeostasis and fracture repair, describing the signaling mechanisms leading to their recruitment at the site of damage and their effective role in bone regeneration. They also highlight new findings about the developmental origin of macrophage and osteoclasts and how a population of EMP-derived embryonic macrophages persist in the adult, acting independently from the HSCs-derived peripheral monocytes.

The concept of the persistence of EMP derived cells in the adult and their key role in physiological and pathological tissue homeostasis is also discussed in the review from Mass and Gentek. Focusing on macrophages and mast cells, they present emerging evidence that demonstrate how the different ontogeny is related to different cell roles. Moreover, in support of the Developmental Origins of Health and Disease theory (DOHaD), they highlight how perturbation of EMP-derived macrophage and mast cells programming and differentiation, due to somatic mutation of early maternal or environmental adverse events, can lead to a wide variety of lifelong diseases in the adult, spanning from allergy to neurological disorders and cancer.

## Erythropoiesis: Subsequent Waves and New Windows For Therapeutic Intervention

Beside myeloid cells, EMPs generate the second embryonic wave of erythroid cells, which acts as a bridge between the first yolk sac-derived Primitive Red Blood cells and the establishment of HSC-dependent adult hematopoiesis. Similar to HSCs, EMPs originate from the hemogenic endothelium, through the endothelial to hematopoietic transition (endoHT).

The receptor tyrosine kinase KIT is a known key regulator of definitive erythropoiesis but it is also expressed in the hemogenic endothelium. This evidence raises the question of its possible functional role in endoHT. Fantin et al. combine mouse genetics and single cell transcriptomic analysis to address this point. Results show that Kit is dispensable for endoHT and for EMPs immediate differentiation. Instead, after EMPs seed the fetal liver, Kit loss significantly reduces EMPs-derived erythropoiesis to the benefit of the alternative myeloid lineage, uncovering the role of Kit in EMPs downstream erythroid commitment.

In humans, the different waves of erythropoiesis are accompanied by the production of different types of hemoglobin: embryonic HbE, fetal HbF and adult HbA. The switch from HbF (α2γ2) to HbA (α2β2) has relevant clinical interest because the persistence of HbF after birth ameliorates β-thalassemia and Sickle Cell Disease (SCD), the most common monogenic diseases worldwide. Barbarani et al. focus on the regulatory networks controlling γ-globin expression in normal and aberrant conditions. Interestingly, in some cases, such as in Hereditary Persistence of Fetal Hemoglobin (HPFH), postnatal γ-globin expression is caused by mutations within the β-locus or within modifiers genes in adult cells. Instead, in the particular case of juvenile myelo-monocytic leukemia (JMML), a rare aggressive pediatric cancer, high HbF together with other fetal red cells traits, suggest a fetal origin of cancer cells, uncovering the cellular heterogeneity underlying the postnatal HbF phenotype.

Early fetal stages of hematopoiesis may represent a potential window for therapeutic intervention for hematological diseases. In their article, Villaverde Cortabarria et al. discuss the feasibility of *in utero* stem cell transplantation (IUSCT) to cure Sickle Cell Disease. Hematopoietic stem cell transplantation (HSCT) is the only SCD cure but it is greatly limited by the availability of matched donors. The option of prenatal intervention, before fetal immune system maturation, could significantly enhance allogenic engraftment, donor-specific tolerance, and lifelong chimerism without immunosuppression. Beside immune response, the host-donor cells competition within the niche represents a second critical obstacle for IUSCT. Despite the scientific, technical, and ethical challenges posed by IUSCT, the perspective to offer a definitive cure for SCD preventing the occurrence of anemia and of major organs damage, warrants further investigation.

## Hematological Malignancies: From Cell of Origin to Treatment

Leukemia is one of the most frequent malignancies in children, dominated by acute lymphoblastic leukemia of the B cell lineage, B-ALL. Even though prognosis has improved markedly the last decades, with survival rates reaching 90%, some subgroups still have dismal prognosis. There is ample evidence that the disease initiates *in utero* and this is the main topic of the review by Cazzola et al. The cell of origin and initiation of the disease, in relation to embryonic hematopoiesis, as well as the need of refined models to study the disease are considered. Moreover, the fact that the disease may initiate in an HSC-independent progenitor, a largely unexplored area of research, is also highlighted.

A minor fraction (10–15%) of pediatric ALL are of T cell origin, a leukemia that usually affect slightly older children than B-ALL. Evidence suggest that T-ALL may also have a prenatal origin, and this is investigated further in the study by Ding et al. When overexpressing Notch pathway, a common mutation in T-ALL, embryonic cells from the paraaortic splanchnopleura (P-Sp) but not from the yolk sac, were shown to give rise to T-ALL when transplanted into mice. Overall the results suggest that there may be a prenatal origin of T-ALL.

In contrast to B-ALL, which has highest incidence in children, acute leukemia of myeloid lineage increases with age and is the most common acute leukemia in adults. Histone deacetylase 8 (HDAC8) has been shown to be overexpressed in some subtypes of Acute Myeloid Leukemia (AML) and its role in normal hematopoiesis as well as possible therapeutic target is the focus of the study by Spreafico et al. By using zebrafish embryos overexpressing Hdac8 an expansion of hematopoietic stem and progenitor cells (HSPCs) was seen. The phenotype could be reversed by an HDAC8 inhibitor, that was shown to induce p53-mediated apoptosis. The HDAC8 inhibitor was also tested in AML cell lines and all together these data indicate HDAC8 as a possible future target for treatment.

Another therapeutic target is the JAK-STAT pathway, a signaling pathway associated with many different hematological malignancies. The role of JAK-STAT in normal as well as malignant hematopoiesis is the topic of the review by Fasouli and Katsantoni. Therapeutical implications, indirect as well as direct inhibition of JAK-STAT pathway and combination with other therapies are also discussed in the review.

## Conclusions

Collectively, our Research Topic highlights that research in hematopoietic development is more active than ever. We think that this field is a perfect example of translation and multidisciplinarity, where discoveries made by basic scientists can bear relevance for clinicians and for the understanding of human disease—and vice versa. It is now clear that cells considered until few yers ago as embryonic-confined transient populations can in fact persist after birth, where they functionally contribute to adult tissues in a range of different ways. We anticipate that this topic will continue to be intensively studied in the upcoming years.

## Author Contributions

All authors equally contributed to the article and approved the submitted version.

## Conflict of Interest

The authors declare that the research was conducted in the absence of any commercial or financial relationships that could be construed as a potential conflict of interest.

## Publisher's Note

All claims expressed in this article are solely those of the authors and do not necessarily represent those of their affiliated organizations, or those of the publisher, the editors and the reviewers. Any product that may be evaluated in this article, or claim that may be made by its manufacturer, is not guaranteed or endorsed by the publisher.

